# Neuroimaging Technology in Exercise Neurorehabilitation Research in Persons with MS: A Scoping Review

**DOI:** 10.3390/s23094530

**Published:** 2023-05-06

**Authors:** Brian M. Sandroff, Caroline M. Rafizadeh, Robert W. Motl

**Affiliations:** 1 Center for Neuropsychology and Neuroscience Research, Kessler Foundation, 1199 Pleasant Valley Way, West Orange, NJ 07052, USA; crafizadeh@kesslerfoundation.org; 2 Department of Physical Medicine and Rehabilitation, Rutgers New Jersey Medical School, Newark, NJ 07103, USA; 3 Department of Kinesiology and Nutrition, University of Illinois Chicago, Chicago, IL 60607, USA; robmotl@uic.edu

**Keywords:** exercise, MRI, brain, rehabilitation, multiple sclerosis

## Abstract

There is increasing interest in the application of neuroimaging technology in exercise neurorehabilitation research among persons with multiple sclerosis (MS). The inclusion and focus on neuroimaging outcomes in MS exercise training research is critical for establishing a biological basis for improvements in functioning and elevating exercise within the neurologist’s clinical armamentarium alongside disease modifying therapies as an approach for treating the disease and its consequences. Indeed, the inclusion of selective neuroimaging approaches and sensor-based technology among physical activity, mobility, and balance outcomes in such MS research might further allow for detecting specific links between the brain and real-world behavior. This paper provided a scoping review on the application of neuroimaging in exercise training research among persons with MS based on searches conducted in PubMed, Web of Science, and Scopus. We identified 60 studies on neuroimaging-technology-based (primarily MRI, which involved a variety of sequences and approaches) correlates of functions, based on multiple sensor-based measures, which are typically targets for exercise training trials in MS. We further identified 12 randomized controlled trials of exercise training effects on neuroimaging outcomes in MS. Overall, there was a large degree of heterogeneity whereby we could not identify definitive conclusions regarding a consistent neuroimaging biomarker of MS-related dysfunction or singular sensor-based measure, or consistent neural adaptation for exercise training in MS. Nevertheless, the present review provides a first step for better linking correlational and randomized controlled trial research for the development of high-quality exercise training studies on the brain in persons with MS, and this is timely given the substantial interest in exercise as a potential disease-modifying and/or neuroplasticity-inducing behavior in this population.

## 1. Introduction

Multiple sclerosis is a common neurological disorder that affects upwards of 1 million adults in the United States and 2.5 million adults worldwide [1]. The disease is characterized by inflammatory processes and multifocal demyelination, followed by transection of axons and neurodegeneration [2]. Current recommendations regarding the diagnosis of MS involve establishing dissemination of MS lesions in time and in space based on technology such as 1.5 T or 3 T magnetic resonance imaging (MRI) [3]. Over time, such central nervous system (CNS) damage results in the accumulation of neurological disability and dysfunction, including physiological deconditioning, mobility disability, balance impairment, and cognitive dysfunction. To date, disease-modifying therapies (DMTs) represent first-line treatments for MS and are highly effective for arresting the disease processes and preventing relapses; DMTs are further associated with preservation of brain volume and reduction in lesion burden over the long-term [4]. Phase-III trials support the benefits of DMTs on the disease process and whole-brain neuroimaging metrics, yet such treatments are ineffectual for restoring functions that are compromised by the MS disease processes. This highlights the importance of considering other approaches for restoring functions that are affected by the disease. One such approach involves exercise training, which (as a form of neurorehabilitation) has been suggested to be the best, and perhaps only way to restore function in MS [5].

Exercise training is a powerful neurorehabilitative approach for restoring functions among persons with MS based on a wealth of evidence supporting its multi-systemic beneficial effects on physical activity/physical fitness, mobility, balance, and perhaps cognitive functioning [6]. Such evidence is largely based on exercise training effects on neuroperformance outcomes. By comparison, there is substantially less evidence supporting the effects of exercise training on MRI outcomes in persons with MS, yet this is necessary for establishing exercise training as a neuroplasticity-inducing or neuroprotective behavior [6,7] within the paradigm of neurorehabilitation. Indeed, the inclusion of selective neuroimaging approaches and sensor-based technology among physical activity, mobility, and balance outcomes in such MS research might further allow for detecting specific links between the brain and real-world behavior. Such research is critical for establishing a biological basis for improvements in functioning [6] and elevating exercise within the neurologist’s clinical armamentarium alongside DMTs as an approach for treating the disease and its consequences [8,9].

**Figure 1:**
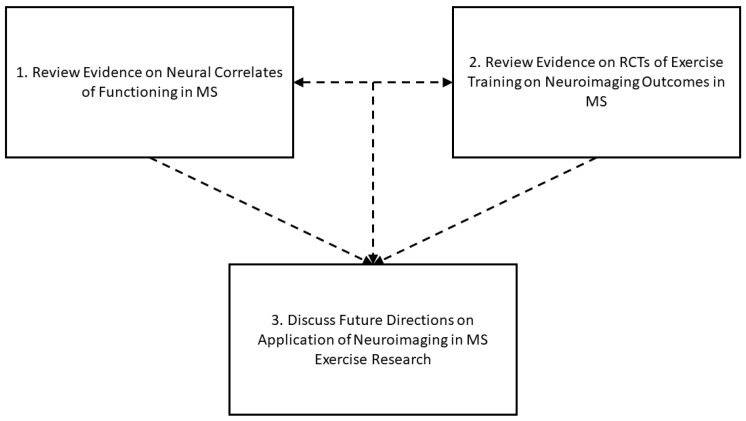
Overview of current scoping review on the application of neuroimaging in MS exercise research.

Given the increased interest in the application of neuroimaging in MS exercise research, the time is ripe to provide a scoping review on this topic as an initial effort to identify available evidence, fieldwide limitations, and provide insight on potential approaches for strengthening the field. To that end, the current paper provides a scoping review on the application of neuroimaging in exercise training research among persons with MS based on searches conducted in PubMed, Web of Science, and Scopus. Scoping reviews provide a broad overview of the available research and compile the main sources and types of evidence available, whereas systematic reviews produce a summary of the efficacy or effectiveness of a given intervention based on a precise set of outcomes [10]. This is important as the drive toward evidence-based practice has accelerated, and new approaches toward evidence synthesis, such as scoping reviews, have emerged in recent years for informing clinical research and practice in chronic diseases and conditions [11]. As depicted in Figure 1, this paper first discusses evidence on the current application of MRI for examining neural correlates of functions that are common targets of exercise training (i.e., physical activity/physical fitness, mobility, and balance). This includes MRI techniques from multiple modalities that address CNS structure (i.e., volumetrics, structural connectivity) and function (i.e., activation, functional/effective connectivity). Notably, although cognitive performance represents a target of exercise training trials in MS [12,13], we do not discuss evidence on neural correlates of cognition in MS for this paper based on a prospective scoping review. As such, the topic has been extensively reviewed [14,15]. This paper next discusses the current application of MRI for capturing neuroimaging outcomes from randomized controlled trials (RCTs) of exercise training in persons with MS. Finally, this paper identifies potential future directions for researchers interested in applying neuroimaging within MS-related exercise research. Such future directions are informed by the evidence on neural correlates of functioning, evidence on neuroimaging outcomes from RCTs of exercise training, and the intersection of the aforementioned correlational and RCT research among persons with MS.

## 2. Neural Correlates of Functions Targeted in Exercise Training Research in MS

There is an emerging corpus of evidence linking neuroimaging outcomes with functional outcomes among persons with MS. Such research underlies the identification of neural targets that might be central for exercise training-related improvements in functions, as well as for developing neurophysiological hypotheses for why and how specific exercise training approaches might induce specific functional adaptations in persons with MS [16]. Such research might further determine prognostic values of neuroimaging outcomes for predicting functional status in MS [17,18]. Using such observational evidence for hypothesis development and subsequent RCT design might be advantageous for steering clear of a generalized search for neural signals within exercise training research that involves neuroimaging in MS [19].

*Neuroimaging Correlates of Physical Fitness and Physical Activity in MS.* We located 15 studies that examine the association among CNS structure/function based on neuroimaging, physical fitness, and physical activity outcomes in persons with MS from our PubMed, Web of Science, and Scopus searches; the studies [20,21,22,23,24,25,26,27,28,29,30,31,32,33,34] are summarized in Table 1. All 15 studies involved MRI as the modality of neuroimaging, though the specific MRI outcomes varied across papers. Indeed, across the 15 studies, the majority (11/15) involved the application of volumetric MRI analyses based on T1-weighted MPRAGE sequences [21,22,23,24,25,27,28,29,32,34], and 5 studies focused on white matter lesion burden based on T2-FLAIR sequences as a neuroimaging outcome of interest [21,23,25,32,34]. This is unsurprising, considering that volumetric and lesion-based outcomes from T1- and T2-weighted sequences, respectively, typically comprise FDA-approved endpoints for trials involving MS DMTs and, further, are important for a definitive MS diagnosis [3]. By comparison, two studies in this category used diffusion-weighted imaging for evaluating associations among structural connectivity outcomes (i.e., fractional anisotropy (FA), mean diffusivity (MD), radial diffusivity (RD), and axial diffusivity (AD)) within white matter tracts [21,26]. Some studies involved functional MRI for measuring BOLD activation during a cognitive task [20] or resting-state functional connectivity (RSFC) [25,31].

Of the 15 studies, 10 identified specific regions of interest (ROI) as correlates of physical fitness and physical activity in persons with MS [22,23,24,25,26,27,28,29,30,31,33]. The majority of these (7/10) focused on subcortical deep grey matter structures (i.e., thalamus, hippocampus, and basal ganglia nuclei [caudate, putamen, pallidum]), and 2 studies focused on the corticospinal tract (CST). Regarding functional outcomes, eight of the studies examined physical fitness [20,21,22,23,24,25,26,27] and eight of the studies examined physical activity (one study examined both physical fitness and physical activity outcomes) [25,28,29,30,31,32,33,34]. The majority of studies that evaluated physical fitness focused on aerobic fitness (i.e., VO_2peak_) [20,21,22,23,24,25], and a minority focused on muscular strength (i.e., isometric peak torque, maximal voluntary contraction) of the lower extremities [26,27]. Regarding physical activity, there was a similar frequency of studies that measured physical activity objectively (i.e., application of motion sensors as a device for measuring steps/time spent in moderate-to-vigorous physical activity; [28,30,31,33]) and subjectively (i.e., with questionnaires; [29,32,33,34]).

Regarding the results from the 15 studies, higher levels of aerobic power, muscle strength, and physical activity were associated with better neuroimaging outcomes (i.e., higher volumes, lower WM lesion burden, greater connectivity/less diffusivity), yet there was heterogeneity in the results, as some studies reported no significant associations among neuroimaging and physical fitness/physical activity outcomes and some results seemingly conflict. For example, two studies reported that better cardiorespiratory fitness based on VO_2peak_ was not associated with higher thalamic volume in persons with MS [22,25]. Yet, one recent study reported a significant association between higher thalamic volume and better cardiorespiratory fitness (based on peak work rate) in MS [24]. This suggests that perhaps the consideration of thalamic volume as a neuroimaging correlate of cardiorespiratory fitness depends upon the specific fitness outcome. Indeed, peak work rate has been hypothesized to better reflect cognitive–motor coupling than VO_2peak_ in this population [24]. Nevertheless, the substantial heterogeneity in neuroimaging modalities/outcome measures and physical fitness/physical activity outcome measures renders the generation of strong conclusions regarding consistent neuroimaging correlates of physical fitness/physical activity in MS difficult.

*Neuroimaging Correlates of Mobility in MS.* Our PubMed, Web of Science, and Scopus searches identified 30 studies that have examined the association among neuroimaging and mobility outcomes in persons with MS; the studies [23,24,27,35,36,37,38,39,40,41,42,43,44,45,46,47,48,49,50,51,52,53,54,55,56,57,58,59,60,61] are summarized in Table 2. Overall, the majority of studies (26/30) used MRI as the primary imaging modality; 3 studies used fNIRS, and 1 study used EEG as the primary imaging modality. Of the 26 studies that adopted MRI paradigms, there was substantial heterogeneity in the specific MRI outcomes. For example, 21 of the 26 studies reported volumetric outcomes, 10 reported WM lesion burden, 9 reported structural connectivity based on diffusion-weighted imaging, and 3 reported RSFC based on fMRI, along with various other approaches (i.e., magnetic resonance spectroscopy, magnetization transfer imaging, BOLD activation during an ankle flexion task, etc.). 

Of the 30 studies, 18 identified specific ROI as correlates of mobility in persons with MS [23,24,27,36,42,46,47,48,50,51,52,54,61]. The specific ROI were highly variable across studies. The most commonly identified ROI involved specific subcortical deep grey matter structures (8/18) and cerebellar regions (7/18). Other ROI involved the spinal cord, corticospinal tract, and motor areas. Regarding the mobility outcomes, 18 of the 30 studies focused on walking speed (e.g., T25FW), 8 focused on walking endurance (e.g., 6MW, 2MW), 8 focused on functional mobility (e.g., TUG, SSST), 6 focused on spatiotemporal parameters of gait (e.g., step length, cadence, double support time measured using sensor-based technology consisting of an instrumented, electronic walkway), and 6 focused on dual-task performance (i.e., walking while simultaneously completing a cognitive task).

Regarding the results, better mobility tended to be associated with better neuroimaging outcomes (i.e., larger volumes, lower WM lesion burden, better structural connectivity, etc.) in persons with MS, yet the pattern of results was heterogenous. Several studies reported no significant associations among neuroimaging and mobility outcomes [23,37,49,61], while some studies reported concurrent associations among specific neuroimaging and mobility outcomes along with no associations among other neuroimaging and mobility outcomes [35,38,39,45,46,50,51,52,54], as well as cases wherein separate studies reported opposing results [23,24]. Further, there was minimal replication of results such that multiple studies reported different findings using different methodological approaches. As such, the substantial heterogeneity in neuroimaging modalities/outcome measures and mobility outcome measures renders the generation of strong conclusions regarding consistent neuroimaging correlates of mobility in MS difficult.

*Neuroimaging Correlates of Balance in MS.* Our PubMed, Web of Science, and Scopus searches identified 13 studies that have examined the association among neuroimaging outcomes and balance outcomes in persons with MS; the studies [57,60,61,62,63,64,65,66,67,68,69,70,71] are summarized in Table 3. All thirteen studies in this area involved MRI as a neuroimaging modality [57,60,61,62,63,64,65,66,67,68,69,70,71], and one study further included somatosensory and motor evoked potentials [65]. Interestingly, the most common MRI outcomes of studies assessing balance involved structural connectivity based on diffusion-weighted imaging (7/13) [60,61,62,63,64,67,69], WM lesion burden (6/13) [57,65,66,68,69,71], followed by volumetric outcomes (4/13) [57,60,68,69]. Fewer studies examined RSFC based on fMRI or used other MRI approaches (e.g., magnetization transfer imaging).

All 13 studies identified specific ROI as potential correlates of balance in persons with MS. The most commonly identified ROI involved the cerebellar regions (9/13) [60,61,63,64,66,68,69,70,71], followed by spinal cord regions (4/13) [57,65,68,69] and specific basal ganglia nuclei (3/13) [61,66,70]. Regarding the balance outcomes, 7 of the 13 studies focused on center of pressure (COP) sway during static posturography paradigms using sensor-based technology consisting of force platforms [57,60,61,66,68,69,71], 4 studies focused on dynamic balance paradigms [62,63,67,70], and 2 others characterized balance based on rating scales [64,65].

Regarding the results, better balance was associated with better neuroimaging outcomes (i.e., better structural connectivity, lower WM lesion burden, larger volumes, better functional connectivity, etc.) in persons with MS. One noticeable trend was that studies that adopted diffusion-weighted imaging paradigms consistently reported significant associations of better structural connectivity within cerebellar regions (i.e., peduncles, lobules) with better balance (i.e., lower COP sway, better dynamic balance) [60,62,63,64,67,69]. Nevertheless, there was still substantial heterogeneity within this area based on a proportion of studies reporting null or conflicting results [61,68,71], as well as differing patterns of selectivity/generality of associations among MRI and balance outcomes [66,70]. Notably, one study did report no associations among cerebellar DTI outcomes and balance outcomes in MS [61]. Collectively, as is the case for the other functional targets of exercise training interventions, it is difficult to generate strong conclusions on neuroimaging correlates of balance in MS, although structural connectivity within cerebellar WM circuits represents a particularly interesting candidate.

*Neuroimaging Correlates of Cognition in MS.* As described earlier, the neuroimaging correlates of cognition in MS have been extensively reviewed [14,15]. Briefly, numerous MRI metrics that are indicative of disease-related damage to CNS structure and function have been linked with cognitive performance outcomes. For example, white matter lesion burden, poor structural connectivity based on DTI, subcortical deep grey matter atrophy, cortical atrophy, and poor resting-state functional connectivity are associated with impairments in cognitive processing speed, learning and memory, and executive functioning in persons with MS [15]. Further, there have been recent hypotheses regarding impairment of neural network dynamics as an underlying cause of MS-related cognitive dysfunction [72]. However, MRI outcomes do not completely predict cognitive impairment in MS, though grey matter atrophy has emerged as the strongest predictor to date [15]. 

## 3. Neuroimaging Outcomes from Exercise Training RCTs in MS

Our PubMed, Web of Science, and Scopus searches identified 14 papers involving RCTs of exercise training effects on neuroimaging outcomes in persons with MS [23,73,74,75,76,77,78,79,80,81,82,83,84,85]; the studies are summarized in Table 4. Notably, there were two instances of secondary analyses from original RCTs [75,79]; thus, study results from those secondary analyses are combined with results from the original RCTs in Table 4. As a result, we summarize findings across 12 RCTs. We further note the publication of four non-RCT studies that evaluated the effects of exercise training on neuroimaging outcomes in persons with MS [86,87,88,89]; those studies have been reviewed elsewhere [90].

Of the 12 RCTs, the majority (8/12) involved aerobic exercise training [23,74,75,78,79,80,81,82,84,85]. The aerobic exercise training interventions involved heterogeneous modalities of exercise (i.e., walking (n = 2); [74,75,84]), multimodal aerobic exercise (n = 2; [82,85]), cycling (n = 1; [80]), running (n = 1; [78,79]), choreographed aerobics (n = 1; [81]), and non-specific aerobic training (n = 1; [23]). Beyond the aerobic exercise training RCTs, one RCT involved progressive resistance training [76], one involved combined exercise training [77], one involved home-based balance training [73], and one involved robotic exoskeleton-assisted exercise training [83]. The interventions ranged from as short as 4 weeks [83] to as long as 48 weeks [85], and took place between 2 and 10 times per week. Notably, 10 of the 12 RCTs involved exercise that took place two–three times per week. Exercise session durations ranged from as short as 15 min per session to as long as over 60 min per session. The majority of exercise training RCTs (i.e., 10 of 12) involved interventions that progressed in terms of intensity over the course of the intervention, consistent with guidelines for exercise prescription [91].

Regarding neuroimaging modalities, 10 of the 12 RCTs involved MRI [73,74,75,76,78,79,80,81,82,84,85]; 1 involved fMRI [83] and 1 involved fMRI and DTI [77]. The most commonly reported imaging outcomes included volumetrics (n = 8; [23,76,78,79,80,81,82,84,85]), structural connectivity based on diffusion-weighted imaging (n = 6; [73,77,78,79,80,82,85]), resting-state functional connectivity based on fMRI (n = 6; [75,77,79,80,83,84]), and lesion burden (n = 4; [23,80,81,82]). Other imaging outcomes included cortical thickness [76,82], viscoelasticity based on magnetic resonance elastography [74], and BOLD activation in response to a motor task [77]. Of the 12 studies, 9 identified specific ROI to examine exercise-specific CNS adaptations [23,73,74,75,77,78,79,82,83,84,85]; 6 of the 9 studies identified whole-brain imaging outcomes (i.e., percent brain volume change, brain parenchymal fraction) as an ROI [23,76,77,78,79,82,85]. Other ROI involved subcortical deep grey matter structures (i.e., hippocampus, thalamus, basal ganglia nuclei) [74,75,78,79,82,83,84,85], along with network-level ROI (i.e., thalamocortical circuits [75,83], sensorimotor network [77], default-mode network [79,84]). Regarding functional outcomes, 8 of the 12 exercise training RCTs included outcomes of physical fitness (i.e., cardiorespiratory fitness, lower extremity muscular strength; [23,74,75,76,78,79,82,84,85]); this is consistent with the definition of exercise training as planned, structured, repetitive physical activity performed for improving or maintaining one or more aspects of physical fitness [92]. By comparison, seven RCTs included mobility outcomes [23,76,77,78,79,81,82,83], two RCTs included balance outcomes [73,77], and six RCTs included cognitive outcomes [74,75,76,78,79,81,83,84]. Notably, 9 of the 12 RCTs evaluated the effects of exercise training on multiple domains of functioning [23,74,75,76,77,78,79,81,82,83,84].

The results are mixed from RCTs regarding exercise training effects on neuroimaging outcomes in persons with MS. This is largely based on the heterogeneity of experimental design, participant characteristics, exercise prescription parameters, along with neuroimaging modalities and outcomes (Table 4). One consistent pattern of results from the RCTs is that exercise training was generally not associated with improvements in whole-brain level volumetric and lesion-related outcomes relative to control conditions [23,76,77,78,79,81,82,85]. However, some RCTs did report exercise-related changes on selective neuroimaging outcomes within specific ROIs [23,73,74,75,76,77,78,79,83,84,85], and others did not [76,77,78,79,80,82,85]. Such selective exercise-related adaptations were inconsistent from study to study and occurred across different imaging outcomes (i.e., volumetrics, structural connectivity/integrity, cortical thickness, resting-state functional connectivity) and regions (i.e., cortex, subcortical deep grey matter structures, resting-state neural networks), whereby there was no robust pattern of results or consistent exercise-related adaptation that was replicated across trials. Moreover, there was no obvious trend regarding RCT results based on MS clinical course, disability status, or associations among changes in neuroimaging and functional outcomes. Thus, as was generally the case for neuroimaging correlates of functioning, it is difficult to render strong conclusions regarding the effects of exercise training on neuroimaging outcomes in persons with MS.

## 4. Discussion/Future Directions

The initial purpose of the present scoping review was to first identify available evidence on the increasing interest in the application of neuroimaging technology in exercise research in persons with MS. Such interest was evidenced by the recent proliferation of studies of neuroimaging correlates of functioning as well as RCTs of exercise training on neuroimaging outcomes that were identified based on our PubMed, Web of Science, and Scopus searches. Our scoping review then sought to identify potential fieldwide limitations in this area. Overall, there was substantial heterogeneity associated with the correlational and RCT evidence on neuroimaging correlates of function in MS and exercise training effects on neuroimaging outcomes in MS, respectively. Within the correlational research, there was not a consistent neuroimaging outcome that could be considered as a potential biomarker for MS-related dysfunction based on consistent associations across domains of function or consistent associations with a singular sensor-based measure. There were, however, correlations among selective neuroimaging outcomes and functional outcomes in persons with MS that were inconsistent from study to study. The overall heterogeneity in results might be a factor of varying methodological approaches; neuroimaging modalities, outcomes, and analyses; functional outcomes; and participant characteristics (i.e., disability status, presence/absence of dysfunction). Collectively, such heterogeneity underscores the importance of replication for better establishing promising associations among neuroimaging and functional outcomes as potential biomarkers of specific MS dysfunction (e.g., walking speed). This represents a major opportunity for strengthening the overall scientific field. Given that a majority of the correlational research has been published in the last 3 years, we acknowledge that the nascence of the field has not permitted such broad-scale replication of effects to date. To better facilitate such replicative research efforts, investigators might pay particular attention to neuroimaging sequences and analytic approaches that are reported in studies as an approach to potentially reduce the aforementioned heterogeneity. This further applies to functional outcomes, given that physical fitness, physical activity, mobility, and balance are not unitary constructs. Nevertheless, future replication efforts in this area might harmonize approaches and thereby promote stronger collaboration among investigators from different laboratory groups, institutions, and countries. Such efforts further could improve the generalizability of results for eventual uptake into clinical practice. Notably, despite the overall heterogeneity, there was some consistency regarding associations among various structural connectivity outcomes of the cerebellum and balance-related outcomes [60,62,63,64,67,68]. Given this specific pattern of results, focusing on the association amongst cerebellar structural connectivity outcomes and MS-related balance dysfunction represents a particularly attractive target for focal research that aims to establish a potential biomarker for balance dysfunction in MS. 

Regarding the RCT evidence, there too was not a singular neuroimaging outcome that was consistently sensitive to the effects of exercise training. There were beneficial effects of different exercise training approaches on selective neuroimaging outcomes that were inconsistent from study to study. This was likely a factor of heterogeneous exercise training interventions; comparison conditions; neuroimaging modalities, outcomes, and analytic approaches; and participant characteristics. Further, such heterogeneity did not permit an evaluation of a potential dose–response effect of exercise training on neuroimaging outcomes in persons with MS. Interestingly, the majority of RCT evidence in persons with MS involved aerobic exercise training, and this is likely based on the much larger volume of evidence of aerobic exercise effects on neural outcomes in older adults from the general population [93]. Despite the overall heterogeneity of RCT results, there were two consistent trends that emerged. First, exercise training was generally not associated with changes on whole-brain volumetric outcomes (i.e., percent brain volume change) and WM lesion burden. Second, of the studies that reported exercise-related improvements on neuroimaging outcomes, several reported improvements were on functional neuroimaging outcomes [75,78,83,84]. Taken together, this pattern of results aligns with the cognitive rehabilitation literature in persons with MS, whereby different cognitive rehabilitation approaches are consistently associated with functional, but not structural MRI changes in persons with MS [13]. Further, this is consistent with previous hypotheses supporting integrative CNS plasticity based on changes in brain function with exercise training in MS [16], and suggests that future research efforts might focus on functional neuroimaging outcomes, potentially using graph theory-based analytical approaches [94], as potential endpoints in exercise trials that seek to provide a biological basis of exercise effects on functioning in this population.

The present scoping review identified nearly 60 studies on neuroimaging correlates of function (not including cognition), yet only 12 RCTs of exercise training effects on neuroimaging outcomes. We note that many studies examined neural correlates of multiple functional outcomes (i.e., mobility and balance outcomes) in MS, whereby a single study could be included in multiple categories (i.e., double-counted) within this scoping review [23,24,27,57,60,61]. The discrepancy between correlational and RCT research affords the opportunity to link those categories of research for providing potential future directions for exercise/neuroimaging research in MS. Although correlational research does not allow for the generation of causative inferences, the initial consideration of correlations among variables can inform hypothesis development and features of a subsequent RCT [95]. For example, initial correlations among selective physical fitness and MRI outcomes in MS might lead to hypotheses pertaining to domains of exercise training inducing neural changes in selective ROI among certain MS groups. Such mechanistic hypotheses would presumably inform the target sample, intervention design, MRI outcomes, and functional outcomes for inclusion in a subsequent trial. Such an approach is commonplace in exercise research in older adults from the general population [95], but seemingly less so in MS. Findings from RCT research might alternatively inform the development of observational research that can examine mechanisms associated with demand characteristics from interventions and/or participant characteristics that led to a certain pattern of results [96,97]. For example, an RCT of combined exercise training on grey matter volume in MS might report non-significant results that could be explained by a number of unknown factors (e.g., exercise prescription, target sample/disability status, neuroimaging approach, etc.). Researchers can perform a subsequent cross-sectional study for examining correlations among different domains of fitness (as surrogates for combined exercise training) and neuroimaging outcomes that can lead to new hypotheses and design elements for a subsequent trial that might have a higher likelihood of success. The success of either approach for linking the correlational and RCT evidence in MS likely depends upon strong collaborations among exercise researchers, neuroimaging researchers, neurologists, and other stakeholders involved in comprehensive MS care. 

To aid in the facilitation of higher quality exercise/neuroimaging research in MS as in the above examples, we consider the degree to which the correlational evidence maps with that from RCTs as identified in the present scoping review. Overall, there was relatively inconsistent overlap between the correlational studies and RCTs based on large heterogeneity across numerous domains, as outlined above. However, there are several common threads among the correlational and RCT studies. The majority of studies involved MRI as the primary imaging modality. This was unsurprising considering the centrality of MRI in MS differential diagnosis, evaluation of disease progression, and prognosis in clinical practice [3]. Relatedly, regardless of functional outcome or exercise training intervention, volumetric MRI measurements and WM lesion burden were the primary MRI outcomes that were included in the studies. This likely reflects a preponderance of questions regarding the functional consequences of the MS disease process, and the extent to which exercise training can modify disease-related CNS damage, given robust evidence of neuroinflammatory lesions and white and grey matter neurodegeneration with MS. Those outcomes further represent endpoints of clinical trials of DMTs in MS. Despite the commonly included outcomes across correlational and RCT studies, the pattern of results regarding various volumetric and WM lesion-related correlates of different functions in MS did not translate to RCT research. Indeed, there is relatively minimal evidence of exercise-related improvements in whole-brain volume, volumes of subcortical deep grey matter structures, or reductions in WM lesion burden in MS [23,76,77,78,79,81,82,85]. Collectively, such a pattern of results and associated hypotheses embody the fieldwide consideration of exercise training as a potential disease-modifying behavior in MS [8] as well as other questions regarding the efficacy of exercise training compared with DMT on whole-brain (or perhaps regional) volume and T2 lesion burden endpoints. However, the current evidence base seems insufficient for generating such interferences at present. 

The correlational and RCT research less frequently involved diffusion-weighted imaging for evaluating structural integrity of WM tracts and fMRI approaches for measuring resting-state functional connectivity. Notably, the most consistent relationship regarding neural correlates of functioning in MS involved associations among cerebellar structural connectivity metrics with various balance outcomes that were observed in six separate studies [60,62,63,64,67,69]. By comparison, only one RCT [73] evaluated the effects of balance training on cerebellar neuroimaging outcomes in MS and reported balance-training-related improvements in cerebellar WM integrity that coincided with improvements in balance itself. The relative consistency of correlational research along with the single RCT highlights the importance of future research to focus on balance training as a potential neuroplasticity-inducing behavior [90]. Interestingly, several RCTs that reported on significant exercise-related changes in neuroimaging outcomes involved improvements in RSFC [75,78,83,84]. RSFC has been identified as an outcome that reflects core neuroplastic changes [98], yet is inconsistently correlated with functional outcomes in persons with MS [99]. However, as the understanding of disrupted functional connectivity as a primary consequence of MS improves [72], exercise research might begin to adopt such MRI outcomes more frequently in both correlational and RCT applications. Importantly, the inclusion of neuroimaging outcomes pertaining to both structural and functional connectivity might more closely align with hypotheses that aim to establish a mechanism-of-action associated with MS functional impairments or exercise-related restoration of function. Collectively, such a pattern of results and associated hypotheses embody the fieldwide consideration of exercise training as a potential neuroplasticity-inducing [90] behavior in persons with MS.

Although not necessarily mutually exclusive, the two different hypotheses pertaining to exercise as a disease-modifying and/or neuroplasticity-inducing behavior in MS likely involve different rationales for including selective neuroimaging outcomes. For example, studies that address exercise training as a potential disease-modifying behavior might include neuroimaging outcomes that are FDA-approved endpoints for DMT trials (i.e., whole-brain volume, T2 lesion volume) using conventional MRI approaches. By comparison, studies that address exercise training as a neuroplasticity-inducing behavior in MS might have considerably more freedom to include neuroimaging outcomes that are specific for a hypothesized mechanism of action (e.g., structural connectivity of the cerebellar peduncles). Some studies did not provide any rationale for the inclusion of specific neuroimaging outcomes, or designated neuroimaging outcomes as exploratory. Interestingly, other studies took a middle ground and provided rationale for non-specific ROI based on previous research in non-MS populations (e.g., [85]). As there is no gold-standard for hypothesis development in this area, such overall heterogeneity in hypotheses represents a potential opportunity for RCT researchers to consider the correlational evidence in MS for better characterizing exercise training as both a disease-modifying and neuroplasticity-inducing behavior. For example, researchers interested in the hypothesis of aerobic exercise training as a neuroplasticity-inducing behavior in MS might consider specific features of cross-sectional research studies for better developing an RCT. There is cross-sectional evidence that better cardiorespiratory fitness (as a surrogate for aerobic exercise training) is associated with better brain activation during a working memory/processing speed task [20], larger GM volume in midline structures, better regional structural connectivity [21], and larger subcortical deep grey matter volumes [22,24] in persons with relatively mild MS disability. This coincides with other cross-sectional evidence indicating that device-measured ambulatory physical activity is associated with larger subcortical deep grey matter volumes [28,33]. Such cross-sectional evidence might converge with RCT research that focused on walking-based aerobic exercise training (as ambulatory physical activity for improving cardiorespiratory fitness) on structural neuroimaging outcomes involving subcortical deep grey matter structures (i.e., hippocampus) [74,75,84]. Future research efforts might consider such an example when developing subsequent RCTs of exercise training on neuroimaging outcomes in MS.

## 5. Conclusions

Collectively, the present scoping review summarized and characterized evidence on neural correlates of function and neuroimaging outcomes in response to exercise training RCTs in persons with MS, with an eye toward informing future research on disease modification and neurorehabilitation. Overall, our PubMed, Web of Science, and Scopus searches yielded a relatively small number of studies, which represents a field-wide limitation. The vast majority of such studies used MRI as the primary neuroimaging outcome, along with neuroperformance measures of physical activity, physical fitness, mobility, balance, and cognition. Overall, there was a large degree of heterogeneity whereby definitive conclusions regarding a consistent neuroimaging biomarker of MS-related dysfunction and consistent neural adaptation to exercise training in MS could not be identified. Such heterogeneity was based on between-study variation in general methodology; neuroimaging modalities, outcomes, and analyses; functional outcomes; and participant characteristics (i.e., disability status, presence/absence of dysfunction). We further note the general lack of replication of results regarding the correlational and RCT evidence. This pattern of heterogeneous results may have been influenced by the small number of studies that were identified by this scoping review, whereby there is not enough research for a definitive convergence of evidence to emerge. Nevertheless, regarding practical implications, the present review provides a first step for better linking correlational and RCT research for the development of high-quality exercise training studies on the brain in persons with MS, given the substantial interest in exercise as a potential disease-modifying and/or neuroplasticity-inducing behavior in this population. Such exercise training studies can study the changes in CNS using existing and new MRI technology, and quantify changes in behavior, physical function, and free-living performance using sensors such as accelerometers, force platforms, and electronic gait mats. Collectively, such research will provide a stronger biological basis for exercise-related changes in functioning among persons with MS.

## Figures and Tables

**Table 1:** Characteristics of Observational Studies on Neural Correlates of Physical Fitness and Physical Activity in Persons with MS.

Construct	Paper	Imaging Modality	n	MS Clinical Course	Disability Status	Functional Outcomes	Imaging Outcomes/ROI	Primary Results
**Physical Fitness**	Prakash et al., 2007 [20]	fMRI	24	RRMS	**EDSS Mean:** 2.6 **Range:** 0–6	**Cardiorespiratory Fitness:** VO_2peak_	**Imaging Outcomes:** Cortical activation during PVSAT **ROI:** N/A	Higher cardiorespiratory fitness associated with greater recruitment of right IFG/MFG and reduced activation of ACC during PVSAT.
	Prakash et al., 2010 [21]	MRI DTI	21	RRMS	**EDSS Mean:** 2.2 **Range:** 0–6	**Cardiorespiratory Fitness:** VO_2peak_	**Imaging Outcomes:** Volumetrics WM lesion burden Structural connectivity (FA) **ROI:** N/A	After controlling for covariates, better cardiorespiratory fitness was associated with lower lesion load. Better fitness was associated with larger grey matter volumes in the right postcentral gyrus and the midline cortical structures (i.e., MFG, ACC, the precuneus. Better fitness was associated with higher FA in left thalamic radiation and right anterior corona radiata.
	Motl et al., 2015 [22]	MRI	35	RRMS PMS	**EDSS Median:** 5.0 **IQR:** 3.5	**Cardiorespiratory Fitness:** VO_2peak_	**Imaging Outcomes:** Volumetrics **ROI:** Hippocampus Thalamus Basal ganglia	Better cardiorespiratory fitness was significantly associated with larger caudate, putamen, pallidum, and hippocampus volumes when controlling for covariates. No association between cardiorespiratory fitness and thalamic volume when controlling for covariates.
	Albergoni et al., 2023 [23]	MRI	61	RRMS PMS	**EDSS Median:** 3.0 **Range:** 0–6.5	**Cardiorespiratory Fitness:** VO_2peak_ HRR	**Imaging Outcomes:** Volumetrics WM lesion burden **ROI:** Insula	Higher HRR was associated with greater lesion volume in left insula.
	Motl et al., 2021 [24]	MRI	62	RRMS PMS	**EDSS median:** 5.0 **IQR:** 3.5	**Cardiorespiratory Fitness:** Peak power output	**Imaging Outcomes:** Volumetrics **ROI:** Basal ganglia Thalamus	Higher peak power output significantly associated with larger thalamic, caudate, putamen, and pallidum volume.
	Romano et al., in press [25]	MRI fMRI	91	PMS	**EDSS median:** 6.0 **IQR:** 2.5	**Cardiorespiratory Fitness:** VO_2peak_ Peak power output **Physical Activity:** Device-measured MVPA, LPA, SB	**Imaging Outcomes:** Volumetrics WM lesion burden RSFC **ROI:** Thalamus Hippocampus Caudate Pallidum Putamen Amygdala Nucleus accumbens	Worse cardiorespiratory fitness was significantly associated with lower normal WM volume, decreased thalamic RSFC with the left ACC, and increased thalamic RSFC with the left calcarine cortex and right lingual gyrus. No association between device-measured physical activity and structural MRI outcomes. Engaging in less MVPA associated with decreased inter-thalamic RSFC. Engaging in less LPA was associated with increased thalamic RSFC with right hippocampus, left lingual gyrus.
	Baird et al., 2018 [26]	MRI DTI	36	NR	**PDDS median:** 4.0 **IQR:** 2.5	**Muscular Strength:** KE and KF strength (isokinetic dynamometry)	**Imaging Outcomes:** Structural connectivity (FA, MD, RD, AD) **ROI:** CST	When controlling for covariates, larger CST FA was significantly associated with summed strength measures.
	Fritz et al., 2017 [27]	MRI MTI DTI	29	RRMS	**EDSS Median:** 4.0 **Range:** 1.0–6.5	**Muscular Strength:** MVC of hip flexion, extension, abduction based on manual muscle testing	**Imaging Outcomes:** MTR **ROI:** CST Brainstem	Quantitative measures of strength and walking were associated with brain CST pathology. Walk velocity was a significant contributor to MTR and FA.
**Physical Activity**	Klaren et al., 2015 [28]	MRI	39	RRMS PMS	**EDSS Median:** 4.5 **IQR:** 2.5	**Physical Activity:** Device-measured MVPA, LPA, SB	**Imaging Outcomes:** Volumetrics **ROI:** Thalamus Basal ganglia Hippocampus	When controlling for covariates, engaging in more MVPA was significantly associated with larger normal GM, normal WM, thalamus, caudate, pallidum, putamen, and hippocampus volumes. No significant associations between LPA, SB, and MRI outcomes.
	Kalron et al., 2020 [29]	MRI	153	RRMS PMS	**EDSS Median:** 2.0 **Range:** 0–6.5	**Physical Activity:** GLTEQ	**Imaging Outcomes:** Volumetrics **ROI:** Hippocampus Amygdala Brain stem Thalamus Nucleus accumbens Caudate	Physically active persons with MS demonstrated significantly larger hippocampal volume than inactive persons with MS. No other differences between groups for other subcortical volumes when controlling for age, biological sex, total cranial volume.
	Block et al., 2023 [30]	MRI	50	RRMS PMS	**EDSS Median:** 4.0 **Range:** 2.5–6.5	**Physical Activity:** Device-measured steps per day from wrist-worn Fitbit	**Imaging Outcomes:** Volumetrics **ROI:** Spinal cord	Engaging in more steps per day was significantly associated with greater spinal cord GM area, total cord area, and cortical volume fraction.
	Prakash et al., 2011 [31]	fMRI	45	RRMS PMS	**EDSS Mean:** 4.1 **Range:** 2–6	**Physical Activity:** Device-measured activity counts per day	**Imaging Outcomes:** RSFC **ROI:** Hippocampus	When controlling for covariates, engaging in more physical activity was associated with greater RSFC between the hippocampus and posteromedial cortex. No associations between physical activity and hippocampal RSFC with other regions.
	Mokhtarzade et al., 2022 [32]	MRI	45	RRMS PMS	**EDSS Median:** 2.0 **Range:** 0–5	**Physical Activity:** Adapted version of historical activity questionnaire	**Imaging Outcomes:** Volumetrics WM lesion burden **ROI:** N/A	No significant differences in brain volume, lesion volume, and lesion number between high- and low-active groups. When controlling for covariates, engaging in more physical activity (total energy expenditure) was associated with larger brain volume, less lesion burden (similar relationship for moderate and vigorous physical activity).
	Negaresh et al., 2021 [33]	MRI MRS	52	RRMS PMS	**EDSS Mean:** 2.1 **SD:** 1.1	**Physical Activity:** Adapted form of historical activity questionnaire Device-measured MVPA, LPA, SB	**Imaging Outcomes:** Volumetrics Metabolite (NAA, MI, Cho) ratios on Cr **ROI:** Hippocampus Thalamus	Whole-brain and hippocampal volumes were significantly larger in highly active vs. inactive/moderately active groups; thalamic volume not significantly different between groups. After controlling for covariates, engaging in more device-measured MVPA, but not LPA, associated with larger thalamic and hippocampal volume, and higher thalamic and hippocampal NAA/Cr ratio; similar pattern of results for self-reported physical activity.
	Grover et al., 2015 [34]	MRI	31	Pediatric MS	**EDSS Median:** 1.5 **Range:** 0–3	**Physical Activity:** GLTEQ	**Imaging Outcomes:** Volumetrics WM lesion burden **ROI:** N/A	Engaging in more strenuous physical activity was significantly associated with lower T2 lesion volume.

Note: 2MW = two-minute walk; 5-STS = 5 chair sit-to-stand; 6MW = six-minute walk; ACC = anterior cingulate cortex; AD = axial diffusivity; BOLD = blood-oxygenation level dependency; Cho = choline; COP = center of pressure; Cr = creatine or phosphocreatine; CSF = cerebrospinal fluid; CST = corticospinal tract; DLPFC = dorsolateral prefrontal cortex; DTI = diffusion tensor imaging; EDSS = Expanded Disability Status Scale; EEG = electroencephalography; ERP = event-related potentials; FA = fractional anisotropy; fMRI = functional magnetic resonance imaging; GLTEQ = Godin Leisure-Time Exercise Questionnaire; GM = grey matter; HbO_2_ = oxygenated hemoglobin; HbR = deoxygenated hemoglobin; HRR = heart rate reserve; IFG = inferior frontal gyrus; imCTSIB = instrumented version of the modified Clinical Test of Sensory Interaction on Balance; IQR = inter-quartile range; LPA = light physical activity; mCTSIB = modified Clinical Test of Sensory Interaction on Balance; MD = mean diffusivity; MEP = motor evoked potentials; MFG = middle frontal gyrus; MI = myo-inositol; MRI = magnetic resonance imaging; MRS = magnetic resonance spectroscopy; MS = multiple sclerosis; MSWS-12 = Multiple Sclerosis Walking Scale-12; MTI = magnetization transfer imaging; MTR = magnetization transfer ratio; MVC = maximal voluntary contraction; MVPA = moderate-to-vigorous physical activity; N = sample size of persons with multiple sclerosis; N/A = not applicable; NAA = N = acetylaspartate; NDGMV = normal deep grey matter volume; NGMV = normal grey matter volume; NR = not reported; NWMV = normal white matter volume; PDDS = Patient-Determined Disease Steps; PFC = prefrontal cortex; PMC = primary motor cortex; PMS = progressive multiple sclerosis; PVSAT = Paced Visual Serial Addition Test; RD = radial diffusivity; RRMS = relapsing-remitting multiple sclerosis; RSEC = resting-state effective connectivity; RSFC = resting-state functional connectivity; SB = sedentary behavior; SEP = somatosensory evoked potentials; SFG = superior frontal gyrus; SMA = supplementary motor area; SPPB = Short Physical Performance Battery; SSST = six-spot step test; T25FW = timed 25-foot walk; TMS = transcranial magnetic stimulation; TUG = timed up-and-go; VO_2peak_ = peak oxygen consumption; WM = white matter.

**Table 2:** Characteristics of Observational Studies on Neural Correlates of Mobility in Persons with MS.

Construct	Paper	Imaging Modality	n	MS Clinical Course	Disability Status	Functional Outcomes	Imaging Outcomes/ROI	Primary Results
**Mobility**	Hubbard et al., 2016 [35]	MRI DTI	69	RRMS PMS	**EDSS Median:** 5.5 **IQR:** 3.4	**Mobility:** T25FW 6MW Gait parameters	**Imaging Outcomes:** Volumetrics Structural connectivity (FA, MD, RD, AD) **ROI:** N/A	AD was significantly correlated with step length. MD was significantly associated with 6MW, T25FW, gait velocity, step time, and step length. FA was not significantly correlated with any of the walking parameters.
	Motl et al., 2021 [24]	MRI	62	RRMS PMS	**EDSS Median:** 5.0 **IQR:** 3.0	**Mobility:** T25FW 6MW	**Imaging Outcomes:** Volumetrics **ROI:** Thalamus Caudate Putamen Pallidum	T25FW and 6MW performance were significantly associated with larger thalamic, caudate, putamen, and pallidum volumes.
	Baird et al., 2022 [36]	MRI	29	RRMS PMS	**EDSS Median:** 4.0 **IQR:** 1.5	**Mobility:** T25FW 6MW	**Imaging Outcomes:** Volumetrics **ROI:** Thalamus Basal ganglia	Among persons with MS, participants’ larger pallidum volume was associated with faster walking speed.
	Albergoni et al., 2023 [23]	MRI	61	RRMS PMS	**EDSS Median:** 3.0 **IQR:** 6.5	**Mobility:** T25FW 6MW	**Imaging Outcomes:** Volumetrics WM lesion burden **ROI:** Insula	No significant associations among 6MW, T25FW performance and MRI outcomes.
	Baird and Motl, 2021 [37]	MRI	31	RRMS PMS	**EDSS Median:** 4.0 **IQR:** 1.5	**Mobility:** SPPB	**Imaging Outcomes:** Volumetrics **ROI:** N/A	No significant associations among SPPB performance, NGMV, NWMV, and CSF volume in persons with MS.
	Mueller et al., 2022 [38]	MRI MRS	15	RRMS	**EDSS Median:** 4.0 **IQR:** 1.0	**Mobility:** T25FW 6MW	**Imaging Outcomes:** Metabolites (NAA, MI, Cho) Brain thermometry **ROI:** N/A	MI in eleven regions correlated with walking speed and MI in twelve regions correlated with walking endurance. Brain temperature did not correlate with mobility measures.
	Preziosa et al., 2022 [39]	MRI fMRI DTI	57	PMS	**EDSS Median:** 6.0 **Range:** 4.5–6.5	**Mobility:** Dual-task walking (reciting alternating letters while walking at self-selected speed)	**Imaging Outcomes:** Volumetrics WM lesion burden Structural connectivity (FA, MD, RD, AD) RSEC **ROI:** N/A	Higher lesion volume, MD of the WM tracts connecting the left caudate with left thalamus, and left thalamus with the left DLPFC, and lower FA of the WM tract connecting the right caudate with right DLPFC predicted higher dual-task walking speed. No significant MRI, fMRI, or DTI predictors of dual-task cost of walking speed.
	Bollaert et al., 2018 [40]	fMRI	29	RRMS PMS	**EDSS Median:** 6.0 **Range:** 2.0–6.5	**Mobility:** T25FW	**Imaging Outcomes:** Volumetrics RSFC **ROI:** N/A	When controlling for covariates, faster T25FW speed was associated with higher RSFC of the left parahippocampal gyrus, left transverse temporal gyrus, right fusiform gyrus, right inferior temporal gyrus, right lingual gyrus, right pericalcarine cortex, right superior temporal gyrus, and right transverse temporal gyrus.
	Chen et al., 2023, [41]	MRI DTI	25	NR	**EDSS Median:** 2.0 **Range:** 1.0–2.5	**Mobility:** Gait parameters assessed during several different tasks, including straight-line walking, circular walking, dual-task walking, triple-task walking TUG	**Imaging Outcomes:** Cortical thickness WM lesion burden Structural connectivity (FA) **ROI:** N/A	Worse TUG performance and slower stride velocity during dual-tasking was significantly associated with thinner cortex in left precuneus and left temporoparietal junction. Worse TUG performance, but not stride velocity, during dual-tasking, was associated with lower FA in corpus callosum, cingulum, bilateral corticospinal tract, and bilateral superior longitudinal fasciculus.
	Cofré Lizama et al., 2022 [42]	MRI	25	NR	**EDSS Mean:** 1.0 **IQR:** 2.0	**Mobility:** Local divergence exponent (i.e., local dynamic stability across multiple planes)	**Imaging Outcomes:** Volumetrics WM lesion burden WM fiber tractography **ROI:** CST Interhemispheric sensorimotor tracts Cerebellothalamic tracts	Worse local dynamic stability at the sacrum, shoulder, and cervical vertebrae was associated with lower fiber density in the CST. Worse local dynamic stability at the cervical vertebrae was associated with lower fiber density in the interhemispheric sensorimotor tracts.
	Nygaard et al., 2021 [43]	MRI DKI	67	RRMS	**EDSS Median:** 2.5 **IQR:** 5.5	**Mobility:** 6MW SSST	**Imaging Outcomes:** Volumetrics Structural connectivity (MD) **ROI:** N/A	Worse performance on the SSST and 6MW was associated with higher MD in the bilateral parietal lobes, cingulate gyri, precuneus, cuneus, SFG, MFG, and unilateral occipital gyrus, and inferior parietal lobule, postcentral gyrus, inferior parietal sulcus, and bilateral superior frontal gyrus, and MFG, respectively. Worse SSST and 6MW performance was associated with lower thalamic volume.
	Mistri et al., 2022 [44]	MRI	106	RRMS	**EDSS Median:** 1.5 **Range:** 1.0–2.0	**Mobility:** T25FW	**Imaging Outcomes:** Volumetrics WM lesion burden **ROI:** N/A	Better T25FW performance was associated with lower lesion burden, higher normalized brain volume, higher NGMV, NWMV, and NDGMV.
	Mamoei et al., 2020 [45]	MRI TMS	49	RRMS PMS	**EDSS:** N/A	**Mobility:** T25FW SSST 5-STS MSWS-12	**Imaging Outcomes:** Volumetrics WM lesion burden MEP (Central motor conduction time; peripheral motor conduction time) **ROI:** N/A	Central motor conduction time was associated with MSWS-12 scores, and T25FW, SSST performance, but not 5-STS. MRI outcomes not associated with MSWS-12, T25FW, SSST, or 5-STS.
	Chaparro et al., 2017 [46]	fNIRS	10	NR	**EDSS Mean:** 3.9 **SD:** 1.6	**Mobility:** SPPB Over ground gait speed	**Imaging Outcomes:** PFC activation HbO_2_ **ROI:** PFC	For older adults with MS, activation levels were significantly higher during dual task conditions when compared with single task relative to age-matched controls. SPPB performance did not predict walking-related increases in activation.
	Hernandez et al., 2016 [47]	fNIRS	8	RRMS	**EDSS Mean:** NR **Range:** 1.0–6.0	**Mobility:** Dual-task walking (walking at self-selected speed and reciting alternate letters of the alphabet)	**Imaging Outcomes:** PFC activation HbO_2_ **ROI:** PFC	Persons with MS demonstrated larger increases in PFC oxygenation during dual-task walking relative to single-task walking compared with controls.
	Saleh et al., 2018 [48]	fNIRS	14	RRMS	**EDSS:** NR	**Mobility:** Dual-task walking (walking at a self-selected speed and subtracting serial 7 s)	**Imaging Outcomes:** Cortical activation HbO_2_ HbR **ROI:** PMC SMA	Persons with MS demonstrated smaller PMC activation during dual-task walking relative to single task walking compared with controls. Within persons with MS, slower walking speed was associated with increased SMA activation.
	De Sanctis et al., 2020 [49]	EEG	13	RRMS	**PDDS Mean:** 1.6	**Mobility:** Dual-task walking (walking on treadmill and completing visual Go/No-Go task)	**Imaging Outcomes:** ERP **ROI:** N/A	Within persons with MS, N2 differentiation associated with Go/No-Go performance was not significantly different between single- and dual-task walking trials.
	Kalron et al., 2020 [50]	MRI	343	RRMS PMS	**EDSS Median:** 2.5 **IQR:** 6.5	**Mobility:** Walk ratio (calculated based on spatiotemporal gait parameters during self-selected walking)	**Imaging Outcomes:** Volumetrics **ROI:** Hippocampus Amygdala Putamen Caudate Pallidum Thalamus Cerebellum Corpus callosum	Better walk ratio was significantly and selectively associated with cerebellum volume, but not volumes of other brain ROI.
	Fritz et al., 2019 [51]	MRI fMRI DTI	18	RRMS	**EDSS Median:** 2.25 **Range:** 1.5–4	**Mobility:** Spatiotemporal gait parameters during single- and dual-task walking (self-selected walking and subtracting serial 3 s) Dual-task TUG (TUG and subtracting serial 3 s) Dual-task walking (walking at self-selected speed and reciting alternating letters of the alphabet)	**Imaging Outcomes:** Volumetrics BOLD activation during ankle flexion/extension WM lesion burden Structural connectivity (FA, MD, RD, AD) **ROI:** SMA V4	Lower SMA activation was associated with slower TUG-cognitive times. Structural imaging measures of SMA interhemispheric connectivity (AD, RD, MD, but not FA) were significantly related to dual-task walking variability. No association between V4 interhemispheric tract diffusivity and dual-task mobility measures.
	Kalron et al., 2018 [52]	MRI	225	RRMS PMS	**EDSS Mean:** 2.6 **SD:** 1.8	**Mobility:** Spatiotemporal gait parameters during self-selected walking	**Imaging Outcomes:** Volumetrics Cortical thickness **ROI:** Hippocampus Putamen Caudate Pallidum Thalamus Cerebellum Cerebral cortex	Gait coefficient of variation was significantly associated with left hippocampus volume and left putamen volume among fallers with MS; no associations between MRI outcomes and gait variability in non-fallers with MS.
	Grothe et al., 2017 [53]	MRI	45	RRMS SPMS	**EDSS Median:** 1.5 **IQR:** 7.5	**Mobility:** T25FW	**Imaging Outcomes:** Volumetrics **ROI:** N/A	Worse T25FW performance was associated with lower GM volume in Larsell’s lobule VI.
	Fritz et al., 2017 [27]	MRI MTI DTI	29	RRMS	**EDSS Median:** 4.0 **Range:** 1.0–6.5	**Mobility:** Walk velocity TUG T25FW 2MW	**Imaging Outcomes:** MTR **ROI:** CST Brainstem	When controlling for covariates, higher walk velocity and 2MW performance were associated with higher MTR and FA of the CST. When controlling for covariates, better T25FW performance was associated with higher FA of the CST.
	Nourbakhsh et al., 2016 [54]	MRI	42	RRMS	**EDSS Median:** 2.0 **IQR:** 5.5	**Mobility:** T25FW	**Imaging Outcomes:** Volumetrics **ROI:** Thalamus Caudate Putamen Pallidum Cerebellar cortex	No significant cross-sectional associations between T25FW performance and GM volumes. Higher baseline thalamic volume predicted less worsening in T25FW performance over 24 months.
	Motl et al., 2016 [55]	MRI	79	RRMS PMS	**EDSS Median:** 3.5 **IQR:** 3.6	**Mobility:** T25FW	**Imaging Outcomes:** Volumetrics WM lesion burden **ROI:** Thalamus Basal ganglia	Faster T25FW speed significantly was associated with larger thalamic, caudate, putamen, and pallidum volumes and smaller WM lesion burden. Thalamic and caudate volume partially explained differences in T25FW speed between persons with MS and healthy controls.
	Motl et al., 2015 [56]	MRI	61	RRMS PMS	**EDSS Median:** 5.5 **IQR:** 3.0	**Mobility:** T25FW 6MW	**Imaging Outcomes:** Volumetrics **ROI:** Thalamus Caudate Putamen Pallidum	When controlling for covariates, larger pallidum and caudate volumes were associated with better T25FW and 6MW performance. Pallidum volume was identified as the strongest correlate of T25FW and 6MW performance.
	Zackowski et al., 2009 [57]	MRI	42	RRMS PMS	**EDSS Mean:** 3.7 **SD:** 2.0	**Mobility:** Walking speed based on fastest walking speed over 20 feet	**Imaging Outcomes:** Volumetrics MTR of CSF WM lesion burden **ROI:** Cervical spinal cord	Faster walking velocity was associated with lower lateral column MTR of CSF.
	Richmond et al., 2022 [58]	MRI DTI	27	RRMS	**EDSS Median:** 3.5 **Range:** 0–4.0	**Mobility:** Spatiotemporal gait parameters based on walking at self-selected speed over 2 min	**Imaging Outcomes:** Structural connectivity (FA, MD, RD, AD) **ROI:** Transcallosal sensorimotor fiber tracts	Better phase coordination index based on gait parameters was associated with better RD and FA of the cingulate motor area, pre-supplementary motor area, dorsal premotor cortex, and primary sensory area. Better phase coordination index was associated with better RD in supplementary motor area and anterior primary motor cortex. Better phase coordination index was associated with better FA in posterior primary motor cortex.
	Fritz et al., 2022 [59]	MRI DTI	29	RRMS	**EDSS Median:** 4.0 **Range:** 1.0–6.5	**Mobility:** Walking velocity SSST T25FW TUG 2MW	**Imaging Outcomes:** Volumetrics Structural connectivity (FA, MD, RD, AD) **ROI:** Cerebellar peduncles Cerebellar lobules I–VIII	Larger cerebellar peduncle and lobular volumes were associated with better T25FW, TUG, and 2MW performance, faster walking velocity. Lower MD and RD of the superior cerebellar peduncles were associated with faster T25FW speed and walking velocity. Lower MD, AD, and RD of the superior cerebellar peduncles were associated with better TUG, SSST, and 2MW performance.
	Ruggieri et al., 2020 [60]	MRI DTI	49	RRMS PMS	**EDSS Median:** 2.5 **Range:** 1.0–5.5	**Mobility:** T25FW	**Imaging Outcomes:** Volumetrics WM lesion burden Structural connectivity (FA, MD, RD, AD) **ROI:** Cerebellar peduncles Cerebellar lobules I–X	When controlling for covariates, worse T25FW performance was associated with global atrophy and atrophy of cerebellar lobule VIIIIb.
	Sbardella et al., 2015 [61]	fMRI DTI	30	RRMS	**EDSS Median:** 2.5 **Range:** 0–4.0	**Mobility:** T25FW	**Imaging Outcomes:** RSFC Structural connectivity (FA, MD, RD, AD) **ROI:** Cerebellum Basal ganglia Corpus callosum	No reported associations among DTI and RSFC outcomes with T25FW performance.

2MW = two-minute walk; 5-STS = 5 chair sit-to-stand; 6MW = six-minute walk; ACC = anterior cingulate cortex; AD = axial diffusivity; BOLD = blood-oxygenation level dependency; Cho = choline; COP = center of pressure; Cr = creatine or phosphocreatine; CSF = cerebrospinal fluid; CST = corticospinal tract; DLPFC = dorsolateral prefrontal cortex; DTI = diffusion tensor imaging; EDSS = Expanded Disability Status Scale; EEG = electroencephalography; ERP = event-related potentials; FA = fractional anisotropy; fMRI = functional magnetic resonance imaging; GLTEQ = Godin Leisure-Time Exercise Questionnaire; GM = grey matter; HbO_2_ = oxygenated hemoglobin; HbR = deoxygenated hemoglobin; HRR = heart rate reserve; IFG = inferior frontal gyrus; imCTSIB = instrumented version of the modified Clinical Test of Sensory Interaction on Balance; IQR = inter-quartile range; LPA = light physical activity; mCTSIB = modified Clinical Test of Sensory Interaction on Balance; MD = mean diffusivity; MEP = motor evoked potentials; MFG = middle frontal gyrus; MI = myo-inositol; MRI = magnetic resonance imaging; MRS = magnetic resonance spectroscopy; MS = multiple sclerosis; MSWS-12 = Multiple Sclerosis Walking Scale-12; MTI = magnetization transfer imaging; MTR = magnetization transfer ratio; MVC = maximal voluntary contraction; MVPA = moderate-to-vigorous physical activity; N = sample size of persons with multiple sclerosis; N/A = not applicable; NAA = N = acetylaspartate; NDGMV = normal deep grey matter volume; NGMV = normal grey matter volume; NR = not reported; NWMV = normal white matter volume; PDDS = Patient-Determined Disease Steps; PFC = prefrontal cortex; PMC = primary motor cortex; PMS = progressive multiple sclerosis; PVSAT = Paced Visual Serial Addition Test; RD = radial diffusivity; RRMS = relapsing-remitting multiple sclerosis; RSEC = resting-state effective connectivity; RSFC = resting-state functional connectivity; SB = sedentary behavior; SEP = somatosensory evoked potentials; SFG = superior frontal gyrus; SMA = supplementary motor area; SPPB = Short Physical Performance Battery; SSST = six-spot step test; T25FW = timed 25-foot walk; TMS = transcranial magnetic stimulation; TUG = timed up-and-go; VO_2peak_ = peak oxygen consumption; WM = white matter.

**Table 3:** Characteristics of Observational Studies on Neural Correlates of Balance in Persons with MS.

Construct	Paper	Imaging Modality	n	MS Clinical Course	Disability Status	Functional Outcomes	Imaging Outcomes/ROI	Primary Results
**Balance**	Richmond et al., 2021 [62]	MRI DTI	27	RRMS	**EDSS Median**: 3.5 **Range**: 0–4.0	**Balance**: Time-to-boundary based on imCTSIB performance	**Imaging Outcomes**: Structural connectivity (FA, MD, RD, AD) **ROI**: Cortical sensorimotor pathway	Lower RD of sensorimotor pathway was associated with shorter time-to-boundary during eyes open imCTSIB conditions on a compliant surface.
	Odom et al., 2021 [63]	MRI DTI	27	RRMS	**EDSS Median**: 3.75 **IQR**: 3.1–4.0	**Balance**: Path length and root mean square of sway based on mCTSIB performance	**Imaging Outcomes**: Structural connectivity (FA, MD, RD, AD) **ROI**: Cerebellar peduncles	Higher FA in inferior cerebellar peduncles was associated with shorter path length during the proprioceptive-based mCTSIB condition. Higher middle cerebellar peduncle RD was associated with longer path length and smaller root mean square of sway during the visual-based balance condition of mCTSIB. Higher inferior cerebellar peduncle RD was associated with longer path length in the vestibular-based balance condition.
	Gera et al., 2020 [64]	MRI DTI	29	RRMS PMS	**EDSS Mean**: 3.1 (mild ataxia) **EDSS Mean**: 3.9 (moderate ataxia)	**Balance**: International Cooperative Ataxia Rating Scale Mini-BESTest	**Imaging Outcomes**: Structural connectivity (FA, MD, RD, AD) **ROI**: Cerebellar peduncles	RD of the inferior cerebellar peduncles was related to postural sway measures during both eyes open and closed. RD of the of the superior cerebellar peduncle was related to postural sway only in stance with eyes open.
	Ruggieri et al., 2020 [60]	MRI DTI	49	RRMS PMS	**EDSS Median**: 2.5 **Range**: 1.0–5.5	**Balance**: Postural sway during static posturography	**Imaging Outcomes**: Volumetrics Structural connectivity (FA, MD, RD, AD) **ROI**: Cerebellar peduncles Cerebellar lobules I–X	Greater postural sway during eyes open conditions was associated with smaller volumes of cerebellar lobules I–IV, smaller total WM volume, and greater WM lesion burden. Greater postural sway during eyes closed conditions was associated with smaller volumes of cerebellar lobules I–IV and greater WM lesion burden.
	Capone et al., 2019 [65]	MRI SEP MEP	38	RRMS PMS	**EDSS Median**: 2.5 **Range**: 0–6.5	**Balance**: Tinetti Scale	**Imaging Outcomes**: Spinal cord functionality (SEP, MEP) WM lesion burden **ROI**: Spinal cord	Higher Tinetti Scale scores were associated with lower cervical WM lesion burden and shorter central somatosensory conduction time.
	Tona et al., 2018 [66]	MRI fMRI	25	RRMS	**EDSS Median**: 3.0 **Range**: 2.0–4.0	**Balance**: COP sway during static posturography	**Imaging Outcomes**: RSFC WM lesion burden **ROI**: Basal ganglia Cerebellar dentate nuclei	Higher RSFC in the left caudate nucleus was associated with shorter COP path.
	Peterson et al., 2017 [67]	MRI DTI	29	RRMS PMS	**EDSS Mean**: 3.5 **Range**: 2.0–4.0	**Balance**: Postural performance in response to multiple trials of balance testing while standing on a hydraulically controlled platform that oscillated in forward and backwards directions	**Imaging Outcomes**: Structural connectivity (FA, MD, RD, AD) **ROI**: Corpus callosum Sensorimotor cortical regions	Temporal improvement in postural performance was associated with RD changes in the corpus callosum and MD changes in the left superior frontal lobe and arcuate fasciculus.
	Prosperini et al., 2014 [68]	MRI	50	RRMS PMS	**EDSS Median**: 2.5 **Range**: 1.0–5.5	**Balance**: COP sway during static posturography	**Imaging Outcomes**: Volumetrics WM lesion burden **ROI**: Cerebellum Spinal cord	When controlling for covariates, greater COP sway during eyes open and eyes closed conditions was associated with lower cerebellar and upper cervical spinal cord volume, worse brainstem and spinal cord lesion burden, but not whole-brain or cerebellar WM lesion burden.
	Prosperini et al., 2013 [69]	MRI DTI	45	RRMS PMS	**EDSS Median**: 2.5 **Range**: 1.0–5.0	**Balance**: COP sway during static posturography	**Imaging Outcomes**: Volumetrics WM lesion burden Structural connectivity (FA, MD, RD, AD) **ROI**: Cerebellum Spinal cord	When controlling for covariates, higher infratentorial WM lesion burden, lower whole-brain FA, and lower total WM volume significantly predicted greater COP sway. When controlling for covariates, worse balance control was associated with lower regional cerebellar volumes.
	Fling et al., 2015 [70]	fMRI	24	RRMS	**EDSS Median**: 4.0 **Range**: 2.0–4.0	**Balance**: Postural performance in response to multiple trials of balance testing while standing on a hydraulically controlled platform that oscillated in forward and backwards directions	**Imaging Outcomes**: RSFC **ROI**: Cerebellum Basal ganglia	Higher RSFC within the corticocerebellar circuit was associated with better baseline postural control. Lower RSFC within the right cortico-striatal loop was associated with better short-term balance adaptation.
	Sbardella et al., 2015 [61]	fMRI DTI	30	RRMS	**EDSS Median**: 2.5 **Range**: 0–4.0	**Balance**: COP sway during static posturography	**Imaging Outcomes**: RSFC Structural connectivity (FA, MD, RD, AD) **ROI**: Cerebellum Basal ganglia Corpus callosum	No reported associations among DTI and RSFC outcomes with COP sway.
	Prosperini et al., 2011 [71]	MRI	31	RRMS PMS	**EDSS Median**: 3.5 **Range**: 2.0–5.0	**Balance**: COP sway during static posturography	**Imaging Outcomes**: WM lesion burden **ROI**: Cerebellum Brainstem Cerebellar peduncles	Worse brainstem WM lesion burden was associated with greater COP sway under eyes open conditions.
	Zackowski et al., 2009 [57]	MRI	42	RRMS PMS	**EDSS Mean**: 3.7 **SD**: 2.0	**Balance**: COP sway during static posturography	**Imaging Outcomes**: Volumetrics MTR of CSF WM lesion burden **ROI**: Cervical spinal cord	Greater COP sway was associated with greater dorsal column MTR of CSF.

2MW = two-minute walk; 5-STS = 5 chair sit-to-stand; 6MW = six-minute walk; ACC = anterior cingulate cortex; AD = axial diffusivity; BOLD = blood-oxygenation level dependency; Cho = choline; COP = center of pressure; Cr = creatine or phosphocreatine; CSF = cerebrospinal fluid; CST = corticospinal tract; DLPFC = dorsolateral prefrontal cortex; DTI = diffusion tensor imaging; EDSS = Expanded Disability Status Scale; EEG = electroencephalography; ERP = event-related potentials; FA = fractional anisotropy; fMRI = functional magnetic resonance imaging; GLTEQ = Godin Leisure-Time Exercise Questionnaire; GM = grey matter; HbO_2_ = oxygenated hemoglobin; HbR = deoxygenated hemoglobin; HRR = heart rate reserve; IFG = inferior frontal gyrus; imCTSIB = instrumented version of the modified Clinical Test of Sensory Interaction on Balance; IQR = inter-quartile range; LPA = light physical activity; mCTSIB = modified Clinical Test of Sensory Interaction on Balance; MD = mean diffusivity; MEP = motor evoked potentials; MFG = middle frontal gyrus; MI = myo-inositol; MRI = magnetic resonance imaging; MRS = magnetic resonance spectroscopy; MS = multiple sclerosis; MSWS-12 = Multiple Sclerosis Walking Scale-12; MTI = magnetization transfer imaging; MTR = magnetization transfer ratio; MVC = maximal voluntary contraction; MVPA = moderate-to-vigorous physical activity; N = sample size of persons with multiple sclerosis; N/A = not applicable; NAA = N = acetylaspartate; NDGMV = normal deep grey matter volume; NGMV = normal grey matter volume; NR = not reported; NWMV = normal white matter volume; PDDS = Patient-Determined Disease Steps; PFC = prefrontal cortex; PMC = primary motor cortex; PMS = progressive multiple sclerosis; PVSAT = Paced Visual Serial Addition Test; RD = radial diffusivity; RRMS = relapsing-remitting multiple sclerosis; RSEC = resting-state effective connectivity; RSFC = resting-state functional connectivity; SB = sedentary behavior; SEP = somatosensory evoked potentials; SFG = superior frontal gyrus; SMA = supplementary motor area; SPPB = Short Physical Performance Battery; SSST = six-spot step test; T25FW = timed 25-foot walk; TMS = transcranial magnetic stimulation; TUG = timed up-and-go; VO_2peak_ = peak oxygen consumption; WM = white matter.

**Table 4:** Characteristics of Exercise Training Randomized Controlled Trials on Neuroimaging Outcomes in MS.

Paper	Imaging Modality	n	MS Clinical Course	Disability Status	Exercise Prescription	Imaging Outcomes/ROI	Functional Outcomes	Primary Results
Prosperini et al., 2014 [73]	MRI DTI	27	RRMS PMS	**EDSS median:** 3.0 **Range**: 1.5–5.0	**Modality:** Home-based, high intensity, task-oriented feedback balance exercise (WBBS) **Duration:** 12 weeks **Frequency:** 5×/week **Time:** 30 min/session **Intensity:** WBBS games automatically progressed in difficulty based on achievement of criterion scores	**Imaging Outcomes:** Structural connectivity (FA, MD, RD) **ROI:** Cerebellar peduncles	**Balance:** COP sway during static posturography	Exercise training was associated with increased FA, decreased RD of cerebellar peduncles relative to waitlist control condition. Exercise training was associated with reduced postural sway under eyes open conditions relative to control. Reductions in postural sway were associated with improvements in structural connectivity.
Sandroff et al., 2017 [74] Sandroff et al., 2018 [75]	MRI MRE fMRI	8	RRMS	**EDSS median:** 3.0 **Range**: 1.5–4.0	**Modality:** Supervised aerobic treadmill walking exercise **Duration:** 12 weeks **Frequency:** 3×/week **Time:** Progressive (15–40 min/session) **Intensity:** Progressive (40–80% HRR)	**Imaging Outcomes:** Viscoelasticity based on MRE RSFC **ROI:** Hippocampus (MRE) Thalamocortical circuit (RSFC)	**Cardiorespiratory fitness:** VO_2peak_ **Cognition:** CVLT-II SDMT	Exercise training was associated with improved hippocampal viscoelasticity, increased thalamocortical RSFC relative to waitlist control condition.
Kjølhede et al., 2018 [76]	MRI	35	Not reported	**EDSS mean:** 2.9 **Range:** 2–4.0	**Modality:** Supervised progressive resistance training of lower and upper extremities **Duration:** 24 weeks **Frequency:** 2×/week **Time:** Not reported **Intensity:** Progressive (3–4 sets of 6–12 reps between 6 and 15 repetition maximum)	**Imaging Outcomes:** Volumetrics Cortical thickness **ROI:** Exploratory	**Lower extremity muscular strength:** MVC of knee extensors and flexors 5-STS **Mobility:** T25FW 2MW **Cognition:** PASAT	No exercise-related changes in whole-brain PBVC, white/grey matter volume, volumes of subcortical structures relative to waitlist control condition. Exercise was associated with improvements in cortical thickness in 19/74 exploratory ROI relative to waitlist control. Exercise was associated with improvements in lower extremity muscular strength, T25FW performance relative to control. Exercise-related increases in cortical thickness of anterior cingular sulcus and gyrus associated with improvements in lower extremity muscular strength.
Tavazzi et al., 2018 [77]	DTI fMRI	26	RRMS PMS	**EDSS median:** 6.0 **Range:** 4.5–6.5	**Modality:** Supervised aerobic treadmill endurance training Supervised progressive resistance training of the lower extremity **Duration:** 4 weeks **Frequency:** 10×/week **Time:** 30–45 min/session **Intensity:** Endurance training: Moderate (below 16–17 on Borg RPE) Progressive resistance training: Progressive (2–3 sets of 10–15 reps)	**Imaging Outcomes:** Structural connectivity (FA, MD, RD) RSFC BOLD signal during plantar dorsiflexion **ROI:** Sensorimotor network (RSFC)	**Balance:** BBS **Mobility:** T25FW 2MW Dynamic Gait Index	No change in any DTI measures for exercise and control conditions. Both groups demonstrated changes in RSFC, activation during dorsiflexion task, and improvements in 2MW and BBS performance, but between-group differences were not reported. No association among changes in task-related activation and functional outcomes; other correlations among changes not reported.
Feys et al., 2019 [78] Huiskamp et al., 2019 [79]	MRI fMRI DTI	29	Not reported	Not reported	**Modality:** Community-based aerobic running training **Duration:** 12 weeks **Frequency:** 3×/week **Time:** Not reported, distance-based **Intensity:** Progressive (1 minute running bouts through running 5 km without interruption)	**Imaging Outcomes:** Volumetrics Structural connectivity (specific measures not reported) RSFC **ROI:** Subcortical deep grey matter structures (volumetrics) DMN (RSFC)	**Cardiorespiratory fitness:** VO_2peak_ **Lower extremity muscular strength:** 5-STS **Mobility:** T25FW 6MW **Cognition:** PASAT DSST WLG SRT 10/36 SPART	Exercise training was associated with increased left pallidum volume relative to waitlist control. No other between-group differences in whole-brain volume, subcortical volumes, structural connectivity outcomes, or hippocampal/DMN RSFC. Exercise training was associated with improved cardiorespiratory fitness, lower extremity muscular strength, and 10/36 SPART performance relative to control. Improvements in 10/36 SPART performance was associated with increased hippocampal-DMN RSFC in the exercise condition.
Stellmann et al., 2020 [80]	MRI DTI fMRI	57	RRMS	**EDSS median:** 1.5 **Range:** 0–3.5	**Modality:** Aerobic cycle ergometer training (intervals) **Duration:** 12 weeks **Frequency:** 2–3×/week **Time:** Up to 60 min **Intensity:** Progressive (tailored based on numerous variables associated with baseline CPET)	**Imaging Outcomes:** Volumetrics WM lesion burden RSFC Structural connectivity (FA) Node connectivity metrics (i.e., hub disruption index) **ROI:** N/A	Functional outcomes data reported in a larger sample in primary analysis paper	No between-group changes in total brain volume, WM lesion burden, or clustering coefficient and node path length. Exercise training was associated with topology-independent (i.e., no significant effect based on hub/non-hub) increases in functional and structural connectivity metrics. No associations among exercise-related changes in functional and structural connectivity outcomes.
Savsek et al., 2021 [81]	MRI	25	RRMS	**EDSS median:** 2.5–3.0 **Range:** 1.0–6.5	**Modality:** Choreographed aerobics **Duration:** 12 weeks **Frequency:** 2×/week **Time:** 60 min/session **Intensity:** 60–70% HRR	**Imaging Outcomes:** Volumetrics WM lesion burden **ROI:** N/A	**Mobility:** T25FW **Cognition:** SDMT CVLT-II BVMT-R	Overall negligible effects of exercise on MRI outcomes and cognitive performance. Weak effect of exercise training on T25FW speed. Mixed results regarding effects of exercise training vs. control on specific brain volumetrics.
Langeskov-Christensen et al., 2021 [82]	MRI DKI	86	RRMS PMS	**EDSS mean:** Intervention (2.7) Waitlist (2.8)	**Modality:** Aerobic cycling/rowing/cross-training (continuous) Aerobic cycling (intervals) **Duration:** 24 weeks **Frequency:** 2×/week (1× continuous and 1× interval) **Time:** 30–60 min/session **Intensity:** Progressive 65–95% HR_max_	**Imaging Outcomes:** Volumetrics Cortical thickness WM + black hole lesion burden Structural connectivity (FA, kurtosis FA, MD, mean kurtosis tensor) **ROI:** Whole-brain Hippocampus Thalamus Corpus callosum Basal ganglia nuclei Upper spinal cord Cortex (DKI) Hippocampus (DKI) Thalamus (DKI) Basal Ganglia (DKI) Corpus callosum (DKI)	**Cardiorespiratory fitness:** VO_2peak_ Peak work rate **Mobility**: 6MW Other outcomes reported in a secondary paper	No difference in percent brain volume change. Exercise-related improvements in cardiorespiratory fitness. No significant changes in MRI, cortical thickness, or DKI variables after Bonferroni correction.
Androwis et al., 2021 [83]	fMRI	10	RRMS PMS	**Ambulation Index median:** 4.0 **Range:** 2–5	**Modality:** Robotic exoskeleton-assisted walking exercise **Duration:** 4 weeks **Frequency:** 2×/week **Time:** 30–45 min/session **Intensity:** Progressive (reduced assistance from robot from session-to-session)	**Imaging Outcomes:** RSFC **ROI:** Thalamus	**Mobility:** TUG 6MW **Cognition:** SDMT	Statistically significant time x group interaction on RSFC between thalamus and ventromedial prefrontal cortex; exercise was associated with increased RSFC compared with control. Exercise was associated with improvements in TUG and SDMT relative to control. Changes in RSFC were moderately associated with changes in TUG, 6MW, and SDMT.
Sandroff et al., 2021 [84]	MRI fMRI	11	RRMS	**EDSS median:** 3.5 **Range**: 2–4.0	**Modality:** Aerobic treadmill walking exercise **Duration:** 12 weeks **Frequency:** 3×/week **Time:** 15–40 min/session **Intensity:** Progressive 40–80% HRR	**Imaging Outcomes:** Volumetrics RSFC **ROI:** Hippocampus	**Cardiorespiratory fitness:** VO_2peak_ Time to exhaustion **Cognition:** CVLT-II OT-SRT BVMT-R 10/36 SPART SDMT	Significant time x condition interaction on normalized hippocampal volume; exercise was associated with preservation, stretching and toning associated with atrophy. Non-significant interaction on hippocampal RSFC using seed-based approach. Large, time x condition interaction on hippocampal-DMN RSFC using independent components analysis approach; exercise associated with reduced RSFC relative to control condition.
Riemenschneider et al., 2022 [85]	MRI DKI	84	Newly diagnosed RRMS	**EDSS mean:** Exercise 1.4 Health education control 1.8	**Modality:** Aerobic cycling, rowing, cross-training, treadmill (unclear) **Duration:** 48 weeks **Frequency:** 2×/week **Time:** 30–60 min/session **Intensity:** Progressive based on mesocycles; 65–95% HR_max_	**Imaging Outcomes:** Volumetrics Structural connectivity (MD, mean kurtosis tensor, FA, kurtosis FA) **ROI:** Whole-brain (volumetrics) Hippocampus (volumetrics, DKI) Thalamus (volumetrics, DKI) Corpus callosum (volumetrics, DKI) Caudate (volumetrics, DKI) Putamen (volumetrics, DKI) Pallidum (volumetrics, DKI) CST (DKI) Cingulate gyrus (DKI)	**Cardiorespiratory fitness:** VO_2peak_ Other outcomes reported in a secondary paper	Exercise training associated with increase in VO_2peak_. No between-group difference for PBVC or BPF or any regional volumes. Exercise was associated with decreased MD in the thalamus, pallidum, CST, and cingulate gyrus relative to control.
Albergoni et al., 2023 [23]	MRI	61	RRMS PMS	**EDSS median:** 4.5 **IQR:** 3.0–6.5	Modality: Aerobic, specific modality not reported Duration: 2–3 months Frequency: 2×–3×/week Time: 30–40 min/session Intensity: Between 55% and 75% HR_max_	**Imaging Outcomes:** Volumetrics WM lesion burden **ROI:** Insula	**Cardiorespiratory fitness:** VO_2peak_ HRR **Mobility:** T25FW 6MW	Aerobic exercise training was associated with improved 6MW distance relative to active control. PBVC, WM lesion burden, and insula volume did not differ between conditions. Aerobic exercise training was associated with increases in grey matter volume in frontotemporal regions and decreased grey matter volume in parieto-occipital regions compared with active control. Within the exercise condition, greater 6MW improvements were associated with less left anterior insula volume loss.

Note: 2MW = 2-min walk; 5-STS = 5 times sit to stand; 6MW = 6-min walk; BBS = Berg Balance Scale; BOLD = blood-oxygen level dependency; BVMT-R = Brief Visuospatial Memory Test-Revised; COP = center of pressure; CPET = cardiopulmonary exercise testing; CST = corticospinal tract; CVLT- II = California Verbal Learning Test-II; DKI = Diffusion Kurtosis Imaging; DMN = default mode network; DSST = Digit-Symbol Substitution Test; DTI = diffusion tensor imaging; FA = fractional anisotropy; fMRI = functional magnetic resonance imaging; HR_max_ = maximum heart rate; HRR = heart rate reserve; IQR = inter-quartile range; MD = mean diffusivity; MRE = magnetic resonance elastography; MRI = magnetic resonance imaging; MS = multiple sclerosis; MVC = maximal voluntary contraction; N = sample size; N/A = not applicable; OT-SRT = Open-trial Selective Reminding Task; PASAT = Paced Auditory Serial Addition Test; PBVC = percent brain volume change; PMS = progressive multiple sclerosis; RD = radial diffusivity; ROI = regions of interest; RPE = rating of perceived exertion; RRMS = relapsing-remitting multiple sclerosis; RSFC = resting-state functional connectivity; SDMT = Symbol Digit Modalities Test; SPART = Spatial Recall Test; SRT = Selective Reminding Task; T25FW = timed 25 foot walk; TUG = timed up-and-go; VO_2peak_ = peak oxygen consumption; WBBS = Wii Balance Board System; WLG = Word List Generation; WM = white matter.

## Data Availability

Data sharing not applicable.
